# Integrated hollow microneedle-optofluidic biosensor for therapeutic drug monitoring in sub-nanoliter volumes

**DOI:** 10.1038/srep29075

**Published:** 2016-07-06

**Authors:** Sahan A. Ranamukhaarachchi, Celestino Padeste, Matthias Dübner, Urs O. Häfeli, Boris Stoeber, Victor J. Cadarso

**Affiliations:** 1 Department of Electrical and Computer Engineering, University of British Columbia, Vancouver, BC V6T 1Z4, Canada; 2 Faculty of Pharmaceutical Sciences, University of British Columbia, Vancouver, BC V6T 1Z3, Canada; 3 Laboratory for Micro- and Nanotechnology, Paul Scherrer Institute, 5232 Villigen PSI, Switzerland; 4 Department of Mechanical Engineering, University of British Columbia, Vancouver, BC V6T 1Z4, Canada

## Abstract

Therapeutic drug monitoring (TDM) typically requires painful blood drawn from patients. We propose a painless and minimally-invasive alternative for TDM using hollow microneedles suitable to extract extremely small volumes (<1 nL) of interstitial fluid to measure drug concentrations. The inner lumen of a microneedle is functionalized to be used as a micro-reactor during sample collection to trap and bind target drug candidates during extraction, without requirements of sample transfer. An optofluidic device is integrated with this microneedle to rapidly quantify drug analytes with high sensitivity using a straightforward absorbance scheme. Vancomycin is currently detected by using volumes ranging between 50–100 μL with a limit of detection (LoD) of 1.35 μM. The proposed microneedle-optofluidic biosensor can detect vancomycin with a sample volume of 0.6 nL and a LoD of <100 nM, validating this painless point of care system with significant potential to reduce healthcare costs and patients suffering.

A number of comprehensive studies on therapeutic drug monitoring (TDM) for antibiotics (i.e., vancomycin, gentamicin), immunosuppressants (i.e., mycophenolic acid, tacrolimus), anticonvulsants (i.e., valproic acid), chemotherapeutics (i.e., carboplatin, methotrexate), and other drugs have found strong correlations in many drug concentrations between interstitial fluid (ISF) and blood/serum^1^,^2^,^3^. As a concrete example, vancomycin (VAN), an antibiotic that acts against gram-positive bacteria, is one of the drugs that have been identified as a candidate for TDM using ISF due to high correlations in drug level between ISF and blood^3^. It is clinically used as a last resort to treat methicillin-resistant *Staphylococcus aureus* infections, which cannot be treated with most other antibiotics^4^,^5^. VAN is administered intravenously, and peak therapeutic levels range between 20–40 μg mL^−1^, with trough levels being 3–10 μg mL^−1^ 3,6. Above therapeutic levels, VAN can cause severe side-effects such as nephrotoxicity (renal failure) and ototoxicity (irreversible deafness)^6^. A number of assay kits are commercially available to monitor the concentration of VAN in patients under treatment. This includes the VANC Flex^®^ cartridge (Siemens Healthcare Diagnostics Ltd., UK), the QMS^®^ Vancomycin (VANCO) assay (Thermo Fisher; Microgenics Corp., Fremont, CA, USA), and the Emit^®^ 2000 Vancomycin assay (Beckman Coulter Inc., Brea, CA, USA). All these tests require the use of large volumes of serum, between 50–100 μL, collected from blood samples (typically > 1 mL) drawn from the patient. This procedure needs to be repeated as frequently as 3–4 times daily to guide therapy and prevent side effects. Not only do these TDM kits result in high costs and use of laboratory equipment, but they also expose the patients to an invasive procedure and require their hospitalization during the whole treatment. The availability of point-of-care TDM systems to be used by the patient directly would thus be extremely valuable and reduce healthcare expenses.

Microneedles have the potential to enable such point-of-care TDM systems. Microneedles are sub-millimeter needle-like structures predominantly aimed for use in minimally-invasive transdermal drug delivery^7^,^8^,^9^,^10^,^11^. Microneedles can rupture the first and toughest layer of the skin, the stratum corneum, to provide direct access for drug delivery into the underlying viable epidermis and the following dermis^12^. Hollow microneedles have also shown potential in the extraction of blood^13^ and ISF^14^ from the skin. ISF surrounds all tissue cells and is present in the skin. ISF is advantageous for biosensing applications since it does not contain any particulates (red blood cells or platelets), and contains at least 5–10 times less protein than blood serum. However, only extremely low volumes (epidermis: 20 nL mm^−2^, dermis: 800 nL mm^−2^)^15^ can be found on the skin, making the process of ISF extraction rather difficult. Previous work demonstrates the possibility of extracting ISF in small amounts of up to 200 nL per microneedle by capillary action over extended periods of time (15–20 min)^14^ or larger volumes (1–10 μL) using a glass microneedle in combination with vacuum suction (27–67 kPa) over 2–10 minutes^16^. Lab-on-a-chip devices with integrated hollow microneedles have been reported for biosensing applications using ISF collected by capillary action^14^,^17^. These devices required transfer of collected ISF out of the microneedle lumen to the analyte detector. This transfer of ISF presents major limitations due to extremely small volumes of accessible ISF in the skin and the slow extraction that can lead to slow sensor response. Therefore, ISF extraction using hollow microneedles combined with integrated biosensing capabilities for extremely low sample volumes would provide significant opportunities for minimally-invasive TDM and diagnostics.

In this paper, we present the integration of a single microneedle^12^ used simultaneously 1) to collect sub-nanoliter volumes by capillary action and 2) to act as a bioreactor with an optofluidic transducer that forms a VAN sensing system in the therapeutic range with samples as small as 0.6 nL. In order to achieve the low limits of detection (LoD), VAN binding is performed during ISF collection in the inner lumen of the microneedle. The lumen is directly connected to a microfluidic system that provides the required chemicals for conducting the bioanalysis after the collection of the analyte, and that quantifies VAN by optical absorbance sensing.

## Results

### Optofluidic biosensing system

**Figure 1:**
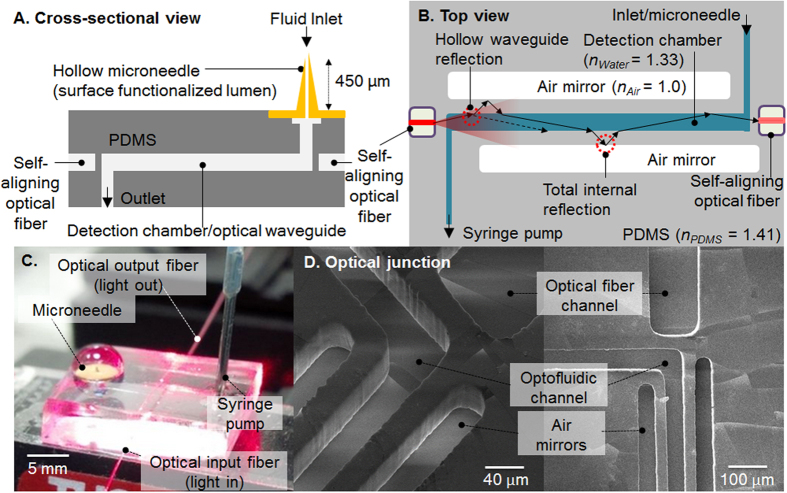
Design and images of the the integrated microneedle-optofluidic biosensor. Cross-sectional schematic view (**A**) and top view design (**B**) of the devices. A surface-functionalized microneedle is integrated to a PDMS optofluidic device equipped with optical fibers. The mechanism of the optical waveguide equipped with air mirrors to guide the incident light from the input fiber through the TMB end-product in the detection chamber to the output fiber using total internal reflection and hollow waveguide reflection is shown in B. An image of the integrated microneedle-optofluidic device during the TMB assay (**C**); and scanning electron micrographs of PDMS optofluidic devices at the junction of air mirrors, microfluidic channel carrying the TMB end-product, and optical fiber self-alignment channels (**D**).

**Figure 2:**
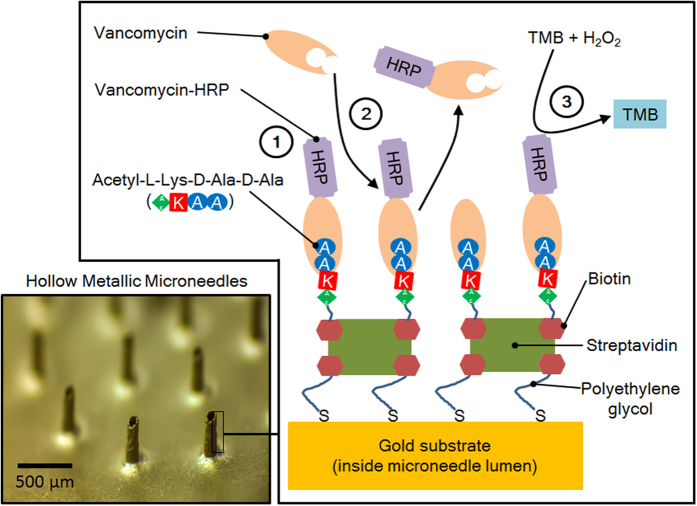
The chemical reaction scheme for the detection of vancomycin in low volume samples inside microneedle lumens. Vancomycin-HRP is pre-loaded to the microneedle surface (1). Vancomycin present in the sample competes for the acetyl-L-lysine-D-alanine-D-alanine binding sites on the microneedle surface and displaces vancomycin-HRP (2). The enzyme-linked TMB assay is used to quantify (in the optofluidic detection chamber/waveguide) the level of bound vancomycin-HRP remaining on the surface (3).

An optofluidic sensing system (Fig. 1) to measure the absorbance of the end-product from an enzymatic reaction that takes place inside the microneedle lumen is implemented. For this purpose, the inner lumen surface is functionalized according to the scheme shown in Fig. 2. The microneedle base surface is attached to a polydimethylsiloxane (PDMS) optofluidic transducer consisting of a detection chamber/optical waveguide, self-aligning optical fibers, and air-mirrors to reduce optical losses, which is fabricated using photolithography and soft-lithography techniques. The use of the alignment sockets allows for the fast and straightforward positioning of the optical fibers directly by hand and without requiring any external or additional equipment. The inlet of the optofluidic transducer is connected to the microneedle base, while the outlet is connected to a syringe pump to improve the flow control for the system characterization. A 5 mm long and 50 μm wide waveguide is selected for the optofluidic sensing device, following optimization with data provided in the Supplementary Material section.

At a 1 mW laser output power into the fiber optics the mean power of noise from the optofluidic waveguide and the optical set-up is ±50 pW, while the output power collected through oxidized 3,3′5,5′-tetramethylbenzidine (TMB) dye in the waveguide ranges between 0.9–43.9 μW. The optofluidic biosensing system thus yields an extremely high signal to noise ratio (SNR) from 42.6 dB (lowest signal recorded) to 59.4 dB (highest signal recorded).

### Surface functionalization of the microneedle device

In the following, a “microneedle base” refers to a 3 mm diameter circular sheet of gold-coated nickel containing a single out-of-plane hollow microneedle projection, with a surface area of the inner lumen of 0.06 mm^2^ and a base surface area of 7.1 mm^2^; the base refers to the side of the metal sheet opposite the protruding microneedle. Surfaces were functionalized either on the backside surface of the microneedle base or inside the microneedle lumen. The streptavidin-biotin interaction has been used widely to build functional surfaces (i.e., inside microneedles in the current study) due to its high affinity binding, stability, and adaptability to many chemical binding schemes. Self-assembled monolayers (SAMs) with 10 mol% biotin-polyethylene glycol-thiol (biotin-PEG-SH) and 90 mol% methoxy polyethylene glycol-thiol (mPEG-SH) formed on gold-coated microneedle surfaces provide the maximum level of streptavidin (Sav) binding of 4 μg mL^−1^ on a microneedle base (on the base surface and lumen combined). Therefore, Sav (4 μg mL^−1^) is used as a stable anchor for subsequent immobilization of drug-specific ligands on microneedle surfaces (i.e., AcKAA peptide for VAN binding; Fig. 2).

### Vancomycin binding assay on microneedle base surface

VAN-HRP conjugate is selectively bound to AcKAA peptides immobilized on the microneedle base surface (Fig. 2) in two steps. A biotin-AcKAA conjugate, formed by linking a biotin N-hydroxysuccinimide (NHS)-ester to AcKAA, is bound to the Sav immobilized on the microneedle base at a concentration of 6.2 mM Biotin-AcKAA. Incubating excessive biotin-AcKAA conjugate on Sav is performed to saturate the biotin-binding sites of the Sav molecules.

**Figure 3:**
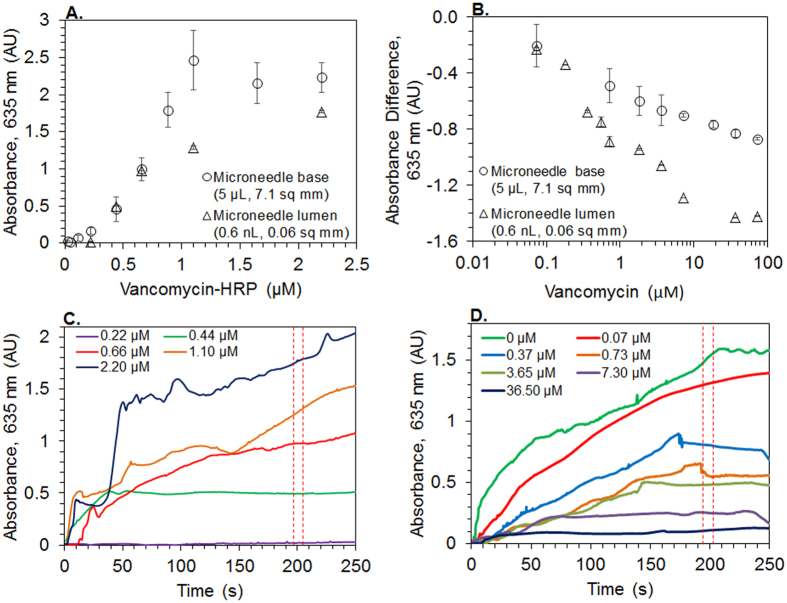
Detection of vancomycin in microliter-to-sub nanoliter volumes in the microneedle-optofluidic biosensor. The vancomycin detection scheme was initially tested on microneedle bases to verify the functionality, prior to transferring to microneedle lumens in the optofluidic device. VAN-HRP binding curve to the microneedle base (5 μL volume, 7.1 mm^2^ area; absorbance measured by NanoDrop ND-1000) and microneedle lumen (0.6 nL volume, 0.06 mm^2^ area; measured by the biosensor from absorbance between 196–205 s from Fig. 3C) (**A**); Vancomycin competitive binding curve to VAN-HRP pre-loaded microneedle base (5 μL volume, 7.1 mm^2^ area; absorbance measured by NanoDrop ND-1000) and microneedle lumen (0.6 nL volume, 0.06 mm^2^ area; measured by the biosensor from absorbance between 196–205 s from Fig. 3D) constructed on a semi-log plot (**B**); absorbance measured with the TMB assay for concentration-dependent VAN-HRP binding to the microneedle-lumen (**C**) and concentration-dependent competitive vancomycin binding to VAN-HRP pre-loaded microneedle lumen (**D**) over time ; (n = 10; error bars represent standard deviations). The reference is normalized to have the maximum absorbance for a concentration of 0 μM vancomycin; hence, increasing the concentration of vancomycin reduces the absorbance in an absorbance sensor (**B**).

The absorbance measurements of a TMB assay (data provided in absorbance units (AU)) following VAN-HRP binding to AcKAA immobilized on the microneedle base surfaces follow a sigmoidal curve over a concentration range from 0.02 to 2.20 μM (Fig. 3A). Strictly monotonic behavior is observed over the range 0.11–1.10 μM VAN*-*HRP (5–50 μg mL^−1^; R^2^ = 0.98), with an average sensitivity of 2.7 AU μM^−1^ VAN*-*HRP. Based on the near-linear range in Fig. 3A, the concentration 0.70 μM VAN*-*HRP is selected for pre-loading the microneedle base for competitive binding with VAN.

The VAN*-*HRP pre-loaded microneedle bases are exposed 10 minutes to VAN concentration in the range from 0.07 to 72.6 μM (corresponding to a 0.1–100 molar ratio of VAN: VAN*-*HRP) for competitive binding. The remaining VAN*-*HRP bound to the surface is quantified using the TMB assay, as shown in Fig. 3B. The semi-log linear detection range for VAN is 0.1–72.6 μM with a detection sensitivity of 0.16 AU/decade VAN concentration. Although it appears that a VAN concentration as low as 0.1 μM can be quantified in the microneedle base surface, the LoD (calculated as three times the blank standard deviation divided by the sensitivity) is determined to be 2.8 μM, due to a high standard deviation (±0.15 AU) of the blank sample (no VAN; only 0.70 μM VAN-HRP present in sample).

### Competitive vancomycin binding assay in extremely low volume microneedle lumens

**Figure 4:**
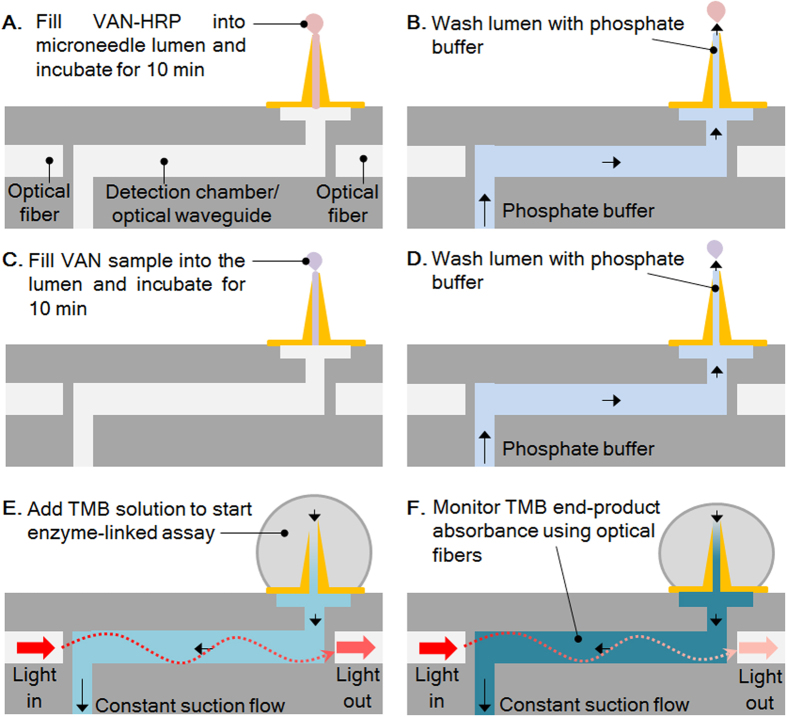
Process of conducting the biosensing process in the microneedle-optofluidic device. Microneedle lumens are pre-loaded with vancomycin-HRP before binding the vancomycin in an unknown sample. The enzyme-linked TMB assay is used to quantify the remaining vancomycin-HRP in the microneedle lumen surface.

Biosensing tests using the microneedle-optofluidic biosensor are conducted according to Fig. 4, where the analyte solutions - by capillary action - are brought in contact with the microneedle lumen only. The biosensing operation process is fully depicted in the Experimental section.

VAN*-*HRP (concentration: 0.22–2.20 μM) is bound to the AcKAA immobilized inside the microneedle lumen (~0.6 nL volume pulled into the lumen). During the TMB assay, the HRP enzyme converts the TMB solution to a colored end-product in a time-dependent manner, as shown in Fig. 3C,D. The increase in the absorbance of the TMB end-product due to increasing concentration of VAN*-*HRP during the course of the TMB assay is continuously monitored by the biosensor (shown in Fig. 3C). A steady state (i.e., plateau of the AU vs. time plot) is reached for most concentration levels of VAN*-*HRP after 150–200 s from the start of the TMB reaction. Absorbance values at 200 ± 5 s are used for further evaluations, including to establish calibration curves (Fig. 3A), as this window presents the best linearity of the calibration curve for the given assay parameters (i.e., TMB solution flow rate and TMB reagent concentrations). A concentration dependent linear increase in binding levels from 0.22–0.70 μM VAN*-*HRP (R^2^ = 0.98) is observed in the microneedle lumens. The sensitivity of the microneedle-optofluidic sensor to detect a change in the VAN*-*HRP concentration is 2.2 AU μM^−1^ VAN*-*HRP. Based on the linear detection range, a VAN*-*HRP concentration of 0.70 μM is again selected for pre-loading the microneedle lumen to achieve a maximum absorbance of ~1 AU from VAN*-*HRP inside the microneedle lumen without competitive binding with VAN.

VAN in a concentration range from 0.07 to 72.6 μM (corresponding to a 0.1–100 molar ratio of VAN: VAN*-*HRP) is competitively bound to VAN*-*HRP pre-loaded microneedle lumen surfaces by capillary action (pulling ~0.6 nL volume into the lumen), simulating the microneedle behavior in real therapeutic applications. The TMB signal decreases for increasing VAN concentrations as shown in Fig. 3B,D following the competitive binding of VAN to the AcKAA peptide inside the microneedle lumen (analyte volume <1 nL) by displacing VAN*-*HRP. Extrapolating from the linear region of the sigmoidal curve in a semi-logarithmic plot in Fig. 3B, a VAN detection range from 0.3–40 μM is determined with a higher VAN detection sensitivity of 0.41 ± 0.03 AU/decade VAN and lower LoD of 84 nM VAN (calculated as the three times the blank standard deviation divided by the sensitivity), compared to microneedle base surfaces.

## Discussion

Microneedle-integrated optofluidic biosensors have an immense potential to tackle most of the challenges that face TDM in one convenient and compact package. In clinical use, the device in this study would eliminate the need for conventional blood-draws using hypodermic needles in TDM patients. At the moment, this device would work directly for TDM drugs that have recently been shown to have a direct correlation between blood and ISF concentration, such as vancomycin, mycophenolate, phenobarbital, methotrexate, and theophylline^3^. By extracting ISF present in the skin, many TDM drugs could be directly analyzed in minimally-invasive and painless procedures with hollow microneedle insertions into the skin and binding the drug inside the microneedle lumens. This is expected to significantly improve patient compliance, speed up TDM, and eliminate anxiety and pain. Furthermore, point of care approaches can be easily envisioned that avoid the use of complex lab equipment to perform TDM, minimize the amount of reagents, and are very user-friendly.

The optofluidic sensing device is optimized to directly perform absorbance measurements of the TMB end-product after exiting the microneedle lumen towards the optical waveguide. The system is specifically designed for the extremely low volume reaction conducted directly inside the microneedle lumen (0.6 nL volume, 0.06 mm^2^ area), where conventional laboratory tools that are used in TDM, such as bench-top spectrophotometers, do not provide sufficient detection and analytical capabilities. The optical detection chamber dimensions are optimized to obtain sufficient light absorbance for the typical high-concentrated TMB end-product (>1 AU). Channel lengths shorter than 5 mm result in lower absorbance values than 1 AU (resulting in lower detection sensitivity). Channel lengths greater than 5 mm deviated from linearity in the calibration curve for absorbance versus channel length due to loss of light from the system. During optical sensing in the selected channel, light is confined in the detection chamber of the optofluidic device as it is designed to simultaneously behave as a waveguide^18^. Additionally, in order to enhance the proper propagation of light with reduced optical losses, air-mirrors are integrated along the detection chamber to assure total internal reflection. Air-mirrors along the selected 5 mm waveguide reduce the intrinsic optical losses from 23 dB without air mirrors to 17 dB (1.7 AU) with air mirrors, improving the SNR significantly.

The gold coating of the microneedle surfaces allow thiol-based functionalization with “ligands” able to trap various target molecules by both direct and competitive binding schemes. The vancomycin trapping and detection scheme described in Fig. 2 involves a combination of chemo-selective gold-thiol surface modification, and high-affinity streptavidin-biotin binding to immobilize and stabilize biotinylated AcKAA ligands on the microneedle surface that attract VAN. Compared to the expensive antibody-based methods of VAN detection performed in clinical settings, including the VANC Flex^®^ cartridge, QMS^®^ VANCO, and Emit^®^ 2000 assay kits, the protocol presented herein is designed with consideration to cost and stability of the surfaces, while maintaining appropriate binding characteristics (i.e., high binding affinity). The compounds used for surface functionalization of microneedle lumens provide flexibility to modify the surface chemistry based on the target-drug candidate, while being low-cost, robust, and stable during long-term storage. Further, the use of a VAN-HRP conjugate for competitively binding to the AcKAA ligands on the microneedles allows for a simple enzyme-linked TMB assay to be used to quantify VAN in the analyte. Similarly, the availability of other peptide/drug candidates with HRP conjugates, such as gentamicin-HRP, makes the proposed biosensing system a platform technology to be used for TDM of a multitude of drug candidates.

The biotin-AcKAA immobilized surface for VAN binding is protected from non-specific binding of analyte and other compounds by a simple bovine serum albumin (BSA) treatment (1 mg mL^−1^ BSA for 30 min), which reduces non-specific binding of VAN*-*HRP on the microneedle base surface significantly (see Supplementary Material). Microneedle surfaces functionalized with only PEG-biotin/Sav show non-specific binding of VAN-HRP (0.7 μM) resulting in TMB end-product absorbance of 0.62 ± 0.05 AU, which is reduced by the BSA treatment (0.04 ± 0.02 AU). Similarly, in the absence of BSA, VAN-HRP (at 0.70 μM) bound to the AcKAA-functionalized surface yields an absorbance of 1.58 ± 0.28 AU, which decreases to 1.00 ± 0.43 AU in the presence of BSA-protection on the surface. Further, microneedle surfaces that are not functionalized with biotin-AcKAA, but are instead functionalized with biotin-BSA show strong prevention of VAN*-*HRP binding (0.04 ± 0.02 AU). Other surface protection strategies explored less successfully to limit non-specific binding of VAN*-*HRP on the surface include binding BSA prior to biotin-AcKAA immobilization and using caseins instead of BSA.

Vancomycin in the VAN*-*HRP conjugate is initially bound to the AcKAA peptide on large microneedle base surfaces to test the binding behavior. The resulting sigmoidal curve (Fig. 3A) can be explained by the relatively low affinity binding between VAN*-*HRP and AcKAA peptide (1–100 μM affinity^19^). Pre-loading of VAN*-*HRP (0.70 μM) onto microneedle base surfaces is performed in a reproducible process to obtain 1.0 ± 0.2 AU during the TMB assay (n = 5). This reproducibility is an important prerequisite to demonstrate the accuracy in quantifying the dynamic displacement of VAN-HRP by unlabelled VAN. According to Kiang *et al*.^3^, VAN concentration in the ISF can vary between 3.2–32 μg mL^−1^ (2.2–22 μM) from peak to trough concentrations, which can be well accommodated by the linear dynamic range for competitive binding of VAN in microneedle bases above the detection limit presented in Fig. 3B (2.8–72.6 μM). The outcome of the competitive VAN binding tests to the microneedle base (7.1 mm^2^ area, 5 μL analyte volume) supports the use of the microneedle lumen only (0.06 mm^2^, 0.6 nL volume) to perform VAN binding and detection.

Analyzing the drug in the microneedle lumen only has advantages over using the microneedle base. The VAN*-*HRP binding curve to the microneedle lumen is similar to the curve for the microneedle base (Fig. 3A), but the LoD for VAN*-*HRP is significantly improved for the microneedle lumen compared to the microneedle base. A larger linear VAN*-*HRP binding range but lower sensitivity are obtained for the microneedle base compared to the microneedle lumen, due to the higher number of AcKAA peptide moieties on the larger microneedle base surface. The highest concentration of VAN*-*HRP that binds to the ligands inside the microneedle lumen before saturation (0.70 μM VAN*-*HRP) is used for pre-loading, which results in a TMB end-product absorbance of 0.98 ± 0.002 AU (similar to the microneedle base). In the microneedle lumen, VAN displaces VAN*-*HRP from the ligands at a significantly higher sensitivity (0.41 AU per decade VAN concentration) compared to the microneedle base (0.16 AU per decade VAN concentration), as shown in Fig. 3B. This increase in sensitivity inside the microneedle lumen is due to lower number of ligands present in the small surface area of 0.06 mm^2^, where every displacement of a VAN*-*HRP from the surface has a significant impact on the resulting concentration of the TMB end-product. As a result of the small area inside the microneedle lumen, the linear VAN detection range decreases from 0.7–72.6 μM (7.1 mm^2^) to 0.3–40 μM (0.06 mm^2^), but it is still able to accommodate the clinically relevant VAN detection range. The LoD for VAN in the microneedle lumen (84 nM VAN) is significantly lower than for the microneedle base, and is the lowest value reported to date. Further, using capillary action to fill microneedle lumens with VAN*-*HRP and VAN ensures that the analyte sample volumes are repeatable, remain solely inside the lumen, and do not come in contact with the microneedle base surface, which can be verified by visual inspection from the backside of the microneedle base.

In comparison, the commercially-available and clinically-used QMS^®^ VANCO assay kit provides a VAN detection range of 1.35–67.3 μM with a sensitivity of 0.77 AU μM^−1^ and a LoD of 1.35 μM; while the Emit^®^ 2000 kit provides a detection range of 1.35–34 μM with a sensitivity of 0.70 AU μM^−1^ and a LoD of 1.35 μM. Both assay kits require 50–100 μL of blood/serum for analysis, but yield significantly higher LoD compared to the microneedle-optofluidic device. With the LoD being orders of magnitude lower than the clinically relevant detection range for VAN and the LoD of other commercial assay kits, the performance of the microneedle-optofluidic device is highly superior to currently used methods for TDM.

The potential of the optofluidic sensing unit to provide real-time rapid detection of surface bound VAN*-*HRP at high sensitivity by measuring the absorbance of less than 40 nL of the TMB end-product at a time using optical fibers (volume capacity of the optical waveguide is 40 nL; experiments performed at a constant flowrate of 10 nL s^−1^) is shown in Fig. 3. In conventional TMB assay protocols performed using bench-top spectrometers, the TMB reaction is allowed to develop the colored end-product for more than 10 min^20^. Due to the small diffusion distances between the TMB reactants and the HRP inside the microneedle lumen, the color development occurs significantly faster to reach saturation (under 200 s for all VAN*-*HRP concentrations). With a high optical SNR between 42.6–59.4 dB, the optofluidic biosensor provides reliable, reproducible, and accurate data during VAN detection and analysis. Features of the optofluidic sensing device, such as the possibility to change waveguide dimensions to improve sensitivity and LoD, adds to the flexibility and adaptability of this platform sensing technology. Finally, the ability of microneedles to collect extremely low volumes of ISF inside their lumens directly, the lack of need for analyte transfer from the collection site to testing site, and the lack of need for microscopes or other sophisticated lab equipment for analysis makes this biosensing platform very portable and easy to use in TDM.

## Conclusions

This first ever integrated microneedle-optofluidic biosensor for medical applications uses surface functionalized gold-coated hollow microneedles. It allows the immobilization of ligands on the inner lumen surface that attract specific drug candidates present in a sub-nanoliter analyte volume. The enzyme assay combined with the optofluidic sensing system for vancomycin detection provides high sensitivity (0.41 AU/decade) and low LoD (84 nM) in clinically relevant ranges (from 0.3–40 μM), for extremely low volume (0.6 nL), and rapid measurements (<5 min in total) of drug binding levels to microneedles. Using vancomycin as a target drug, the potential of the microneedle-optofluidic biosensor for TDM in ISF was demonstrated for point of care applications using a minimally invasive sample extraction and limited need for external equipment (only a diode laser and a photodetector were required). These results prove the potential of the proposed integrated sensors for the development of portable devices that can be used by patients to perform TDM with many drug candidates present in ISF.

## Methods

### Materials

Biotin-polyethylene glycol-thiol (biotin-PEG-SH, mol. wt. 5 kDa, purity >95%) and methoxy-polyethylene glycol-thiol (mPEG-SH, purity >95%) were purchased from Nanocs Inc. (Boston, MA, USA). The acetyl-lysine-d-alanine-d-alanine (AcKAA, mol. wt. 330.38 Da) peptide (purity ≥95% HPLC), vancomycin hydrochloride (100 mg mL^−1^ in DMSO, 0.2 μm filtered, mol. wt. 1449.25 Da), Streptavidin from *Streptomyces avidinii* (lyophilized powder, mol. wt. 60 kDa), 3,3′5,5′-tetramethylbenzidine (TMB, purity ≥95% NT, mol. wt. 240.34 Da), and biotin-labelled bovine serum albumin (biotin-BSA; 8–16 mol biotin per mol BSA) were purchased from Sigma-Aldrich (Buchs, Switzerland). The vancomycin-horseradish peroxidase conjugate (VAN-HRP, 1 mg mL^−1^, mol. wt. 45 kDa) was purchased from Cal Bioreagents (San Mateo, CA, USA); hydrogen peroxide (30% H_2_O_2_, Perhydrol^®^) was purchased from Merck (Darmstadt, Germany); the EZ-Link sulfo-NHS-LC-biotin was purchased from ThermoFischer Scientific (Waltham, MA, USA); and the streptavidin-horseradish peroxidase (Sav-HRP) conjugate (2.5 mg mL^−1^, mol. wt. 110 kDa) was purchased from Invitrogen Corporation (Camarillo, CA, USA). SU-8 2075 photocurable polymer resist and the propylene glycol methyl ether acetate (PGMEA) developer were purchased from MicroChem (Newton, MA, USA); and the polydimethylsiloxane (PDMS) kit (Sylgard 184) was purchased from Dow Corning Corp (Midland, MI, USA).

### Microneedle fabrication

Single hollow metallic microneedle bases were fabricated according to Mansoor *et al*. with minor modifications, which involved a 3-step metal electrodeposition process (gold-nickel-gold electrodeposition)^7^. The microneedles were tapered toward their tip with a height of 450 μm, a tip diameter of 30 μm, and a base diameter of 50 μm. Based on these dimensions, the area and volume occupied inside a microneedle lumen were 0.06 mm^2^ and 0.6 nL, respectively.

Microneedle bases (circular pieces, 3 mm diameter, and 7.1 mm^2^ area) with one needle in the center were cut from the fabricated samples using a metal punching tool.

### 3,3′5,5′-Tetramethylbenzidine assay

The TMB assay is an enzyme-linked assay that is used to quantify the activity of the HRP enzyme. HRP oxidizes 3,3′5,5′-tetramethylbenzidine in the presence of hydrogen peroxide to yield a blue product, which can be detected at a wavelength of 635 nm^20^. In this study, Sav-HRP and VAN*-*HRP conjugates were used to quantify binding concentrations of molecules to the respective surfaces. The absorbance of the TMB oxidation product correlated to the concentration of HRP-conjugate present on a surface and thus the concentration of analyte bound (i.e., Sav-HRP or VAN*-*HRP).

The TMB assay reagent concentrations were optimized to provide a maximum absorbance of 3.0 AU throughout the study. The TMB assay consisted of two solutions: Solution A contained TMB at 0.4 mM, prepared by dissolving 0.1 g of TMB in 2 mL acetone and 18 mL of methanol. Solution B was the TMB reaction buffer solution, which was prepared by dissolving 22.06 g citric acid monohydrate (0.1 M), 5.6 g potassium hydroxide (0.1 M), and 20 μL of 30% hydrogen peroxide (0.4 mM) in 500 mL of milliQ water. Immediately prior to conducting the TMB assay, 50 μL of solutions A and 1 mL of solution B were pre-mixed to obtain the TMB stock solution.

After binding a HRP-conjugate to a microneedle base, a 10 μL volume of the TMB stock solution was placed on top of the microneedle base’s functionalized surface, and incubated for 10 min at room temperature to allow the HRP to produce the TMB colored end-product. After 10 min, a 2 μL volume of the TMB solution was extracted from the microneedle base surface, and its absorbance at 635 nm was determined using a NanoDrop ND-1000 spectrometer (ThermoFischer Scientific, Waltham, MA, USA).

### Microneedle surface functionalization

#### Surface cleaning

All surfaces were cleaned using UV irradiation at a 170 nm wavelength for 10 min using a flat excimer Ex-Mini source (Hamamatsu, Japan). The surfaces were maintained at a 10 mm distance from the source of irradiation during the cleaning process.

#### Self-assembled monolayers

Thiolated and biotinylated PEG chains were employed to form SAMs on gold surfaces^21^,^22^. The molecular weight of the PEG, 5000 Da, was selected due to its ability to reduce non-specific adsorption of proteins in biological fluids^23^. Biotin-PEG-SH and mPEG-SH were prepared fresh by dissolving in milliQ water and 2 vol% ethanol (95% purity) to a concentration of 1 mM. Biotin-PEG-SH and mPEG-SH were mixed to obtain molar fractions ranging from 0–100% biotin-PEG-SH/mPEG-SH to determine the optimum surface density of biotin on the gold-coated substrates. Droplets of 5 μL from each biotin-PEG-SH solution were placed on a glass slide coated with Parafilm, and gold-surfaces brought into contact with the droplets for 2 h at room temperature to form SAMs. The gold-surfaces were withdrawn from the biotin-PEG-SH droplets, and washed three times with 50 mM phosphate buffered saline (PBS, pH 7.4).

#### Streptavidin-biotin linking

The Sav-HRP stock solution (2.5 mg mL^−1^) was diluted with PBS buffer to a concentration of 10 μg mL^−1^ (0.1 μM). Droplets of 5 μL were placed on a Parafilm-coated glass slide. PEG-modified gold substrates were brought into contact with the droplets and incubated for 1 h at room temperature, followed by three PBS washing steps. The binding of Sav-HRP was quantified using the TMB assay. Findings from the Sav-HRP tests were used in the binding of free Sav to biotin to further functionalize the gold-surfaces for vancomycin binding.

Microneedle base inner surfaces containing a SAM of 10% molar ratio of biotin-PEG-S: mPEG-SH were incubated with 5 μL droplets of free Sav (2.4 μg mL^−1^, 0.1 μM) on Parafilm-coated glass slides for 1 h at room temperature, to allow the high-affinity binding of Sav to biotin. At the end of the incubation period, the microneedle bases were removed from the incubation slides, and washed three times with PBS.

#### Vancomycin-HRP binding

A sulfo-NHS-LC-biotin linker (6.24 mM, 4.18 mg mL^−1^) was mixed with a 6.24 mM AcKAA in PBS (2.04 mg mL^−1^) at a volumetric ratio of 1:1, and incubated for 30 min at room temperature to produce the biotin-AcKAA conjugate. Microneedle bases functionalized with Sav were incubated with 5 μL of the 6.24 mM Biotin-AcKAA conjugate for 1 h at room temperature to immobilize the surface ligand to promote vancomycin binding (Fig. 2). The microneedle base surfaces were washed with PBS buffer three times.

To minimize non-specific binding of VAN-HRP to the microneedle base surface and to promote binding to the AcKAA peptides, the surfaces were further treated with BSA (1 mg mL^−1^) for 30 min at room temperature. The microneedle base surfaces were washed with PBS buffer three times.

VAN-HRP concentrations ranging from 0.02–2.20 μM (1–100 μg mL^−1^) were made from a stock solution at 22 μM (1 mg mL^−1^). Each VAN*-*HRP concentration level was tested on five microneedle bases (n = 5) that were functionalized with the AcKAA peptide to determine the VAN*-*HRP binding behavior as a function of concentration. A 5 μL volume of each VAN*-*HRP solution was incubated on the microneedle base for 10 min at room temperature, allowing VAN*-*HRP to bind to the AcKAA peptide. At the end of the incubation, the microneedle bases were washed with PBS and tested using the TMB assay to quantify the bound VAN*-*HRP content. From the absorbance data obtained, a calibration curve (absorbance versus VAN*-*HRP concentration) was constructed for VAN*-*HRP binding to the microneedle base at a 7.1 mm^2^ area.

#### Vancomycin binding

In microneedle bases that were incubated with VAN*-*HRP, a subsequent binding of VAN at concentrations ranging from 0–72.6 μM was performed. VAN concentrations ranging from 0.07–72.6 μM (0.11–105 μg mL^−1^) were made from a stock solution at 69 mM (100 mg mL^−1^). A 5 μL volume of each VAN concentration level was incubated on the microneedle base surface for 10 min at room temperature, allowing VAN to bind to unoccupied AcKAA peptides on the surface or displace bound VAN*-*HRP from the AcKAA peptides. At the end of the incubation, the microneedle bases were washed with PBS and tested using the TMB assay to quantify the effect of VAN concentration on the competitive displacement of bound VAN*-*HRP from the microneedle base. From the absorbance data measured during the TMB assay, a VAN competitive binding curve was constructed for microneedle bases at a 7.1 mm^2^ area.

### Preparation of masters and molds for the optofluidic devices

Two masters (a master 1 for the optical waveguide and fiber alignment, and master 2 for the integration of surface functionalized microneedle bases to the optofluidic devices) were fabricated with an SU-8 negative photoresist using photolithography according to manufacturer recommendations with minor modifications. SU-8 2075 was spin-coated on a SiO_2_-coated silicon wafer at 1700 rpm for 35 s to obtain a 150 μm thick layer for master 1. Following a soft-baking step at 95 °C for 30 min, the SU-8 was exposed to UV light (240 mJ cm^−2^) through a photomask. An immediate post-exposure baking step was conducted directly at 95 °C for 5 min, followed by SU-8 development in PGMEA for 12 min.

Master 2 was fabricated using two SU-8 2025 layers aligned on top of each other for microneedle placement and attachment to the optofluidic device. Layer 1 was spin coated to obtain a 50 μm feature height (1750 rpm), followed by a soft-baking step at 65 °C for 3 min and 95 °C for 9 min. Layer 1 was exposed to UV light (240 mJ cm^−2^) through a photomask. An immediate post-exposure baking step was conducted at 65 °C for 2 min and at 95 °C for 7 min. Layer 2 was spin coated to obtain a 20 μm feature (4000 rpm) and soft-baked on top of layer 1 at 65 °C for 3 min and 95 °C for 6 min; and exposed to UV light (240 mJ cm^−2^) through a photomask with precise alignment to the first exposure. Another post-exposure baking step was conducted at 65 °C for 1 min and at 95 °C for 6 min, followed by SU-8 development in PGMEA for 10 min.

A Sylgard 184 PDMS kit was used to weigh and mix thoroughly 30 g of its base solution to 3 g of its cross-linking agent (10:1 w:w base to cross-linker ratio). The PDMS mixture was degassed under vacuum, poured onto the SU-8 masters, and cured at 80 °C for 1 h to obtain 3 mm thick PDMS slabs with transferred structural features. Once peeled off of the masters, PDMS replicates from master 1 and master 2 were aligned and bonded to each other using PDMS catalyst bonding, according to Samel *et al*.^24^ to securely seal the optofluidic channels and prevent leaking.

### Optofluidic sensor setup and optimization

The overall design of the optofluidic device is outlined in Fig. 1. Waveguide dimensions tested included a height of 150 μm, widths of 50 μm and 100 μm, and lengths ranging from 100 μm to 10 mm. The optofluidic devices consisted of self-aligning optical fiber channels and air-mirrors to confine the light in the sensing region (waveguide) and increase the signal to noise ratio.

Light was coupled in and out of the optofluidic devices by means of input and output fibers, which were integrated by using self-aligning PDMS fiber channels. This allowed to accurately place both fibers without requiring any special, time consuming or expensive processes, at a fixed distance of 30 μm from the integrated waveguide. A 635 nm working wavelength luminescent diode (LED) laser, coupled to a 4 μm diameter single-mode optical fiber (input fiber), was employed as light source for optical biosensing. Light was injected at 1 mW power into the optical waveguide using the input fiber. A 50 μm diameter multi-mode fiber (output fiber) connected to a silicon PIN photodiode was placed on the opposite end of the waveguide (at a distance of 30 μm from the waveguide) and used to collect the optical output light power from the waveguide. A PM100D compact power and energy meter console (Thorlabs, Newton, NJ, USA) was used to acquire the optical output power data signal at 1 Hz frequency.

Characterization of optical properties of the devices was performed using solutions of methyl green, which absorbs light at 635 nm (similar to the TMB assay end-product), diluted to an absorbance reading of 2.5 AU per 1 cm path length.

### Integration of microneedle to optofluidic sensor

The area surrounding the microfluidic inlet, where the surface-functionalized microneedle base was attached, was first treated for a few seconds with an atmospheric-pressure helium plasma to decrease the surface contact angle. The microneedle base was aligned with the microfluidic inlet and placed on the PDMS surface, followed by application of an instant-bonding cyanoacrylate adhesive around the circular microneedle base perimeter. The adhesive completely cured within 10 min, allowing the use of the microneedle-integrated optofluidic device for biosensing experiments.

### Performance of the microneedle-optofluidic sensor

Biosensing tests using the microneedle-optofluidic biosensor were conducted according to Fig. 4. Microneedle tips of integrated biosensor devices equipped with optical fibers were brought in contact with 1 μL droplets of 0.22–2.20 μM VAN*-*HRP on a Parafilm-coated glass slide for 60 s, allowing the microneedle lumen to fill via capillary action (Fig. 4A). VAN*-*HRP was incubated inside the microneedle lumen for 10 min at room temperature, followed by washing the microneedle lumen with PBS (Fig. 4B). During the washing step with PBS, the optical output power collection process was initiated to obtain a baseline stabilized optical readout for PBS flow through the optical waveguide and microneedle lumen. A 50 μL droplet of the TMB stock solution was placed on the microneedle base. A suction-flow of 10 nL s^−1^ was established through the microneedle and the waveguide using a syringe pump that was attached to the fluid outlet of the biosensor. The TMB stock solution was flown through the biosensor for 300 s, and afterwards replaced with 50 μL of PBS to wash away any unreacted TMB stock solution from the biosensor.

Microneedle lumen were pre-loaded with 0.70 μM VAN*-*HRP; and subsequently microneedle tips were brought in contact with 1 μL droplets of VAN ranging from 0.07–72.6 μM on a Parafilm-coated glass slide for 60 s, allowing the microneedle lumen to fill via capillary action (Fig. 4C). VAN was incubated inside the microneedle lumen for 10 min at room temperature, followed by washing the microneedle lumen with PBS (Fig. 4D). As done previously, a 50 μL droplet of the TMB stock solution was placed on the microneedle base and a suction-flow of 10 nL s^−1^ was established through the microneedle and the waveguide to conduct the TMB assay in the biosensor (Fig. 4E,F). From the data collected, average absorbance (at 635 nm) of the TMB end-product was determined in the time from 196 s to 205 s.

## Additional Information

**How to cite this article**: Ranamukhaarachchi, S. A. *et al*. Integrated hollow microneedle-optofluidic biosensor for therapeutic drug monitoring in sub-nanoliter volumes. *Sci. Rep.* **6**, 29075; doi: 10.1038/srep29075 (2016).

## Supplementary Material

Supplementary Information
