# Supplementary material for: Integrated hollow microneedle-optofluidic biosensor for therapeutic drug monitoring in sub-nanoliter volumes
Source: Sci Rep. 2016 Jul 6;6:29075. doi: 10.1038/srep29075 (PMC4933911; doi:10.1038/srep29075)
Supplement: Supplementary Information [file srep29075-s1.pdf]

Integrated hollow microneedle-optofluidic biosensor for therapeutic drug monitoring in sub-nanoliter volumes

Sahan A. Ranamukhaarachchi[a,b,c], Celestino Padeste[c] , Matthias Dübner[c], Urs O. Häfeli[b], Boris Stoeber[a,d], Victor J. Cadarso[c,*]

[a] Department of Electrical and Computer Engineering, University of British Columbia, Vancouver, BC Canada V6T 1Z4

[b] Faculty of Pharmaceutical Sciences, University of British Columbia, Vancouver, BC Canada V6T 1Z4

[c] Laboratory for Micro- and Nanotechnology, Paul Scherrer Institute, 5232 Villigen PSI, Switzerland

[d] Department of Mechanical Engineering, University of British Columbia, Vancouver, BC Canada V6T 1Z4

Corresponding author:

Victor J. Cadarso
Laboratory for Micro- and Nanotechnology, Paul Scherrer Institute,
ODRA/119, 5232 Villigen PSI
Switzerland
Email: victor.cadarso@psi.ch
Tel: +41 56 310 5146
Fax: +41 56 310 2646

Supplementary Material

Optimization of the detection chamber/optical waveguide dimensions (channel length and width) using diode laser input light and methyl green dye (model dye for TMB end-product, absorbing at 635 nm) showed a linear relationship between the absorbance and the channel length independent of the channel width (**Figure *S*1A**). **Figure *S*1B** demonstrates the impact of blocking the AcKAA-functionalized surface with BSA to prevent non-specific binding of VAN-HRP at a concentration of 0.70 μM. The waveguide with 5 mm length and 50 μm width dimensions provided an absorbance measurement of over 1 absorbance unit (AU) within the observed linear calibration range for a typical TMB end-product. Beyond ~1.3 AU and ~5 mm channel length, absorbance values deviated from the linear range of the waveguide dimensions.

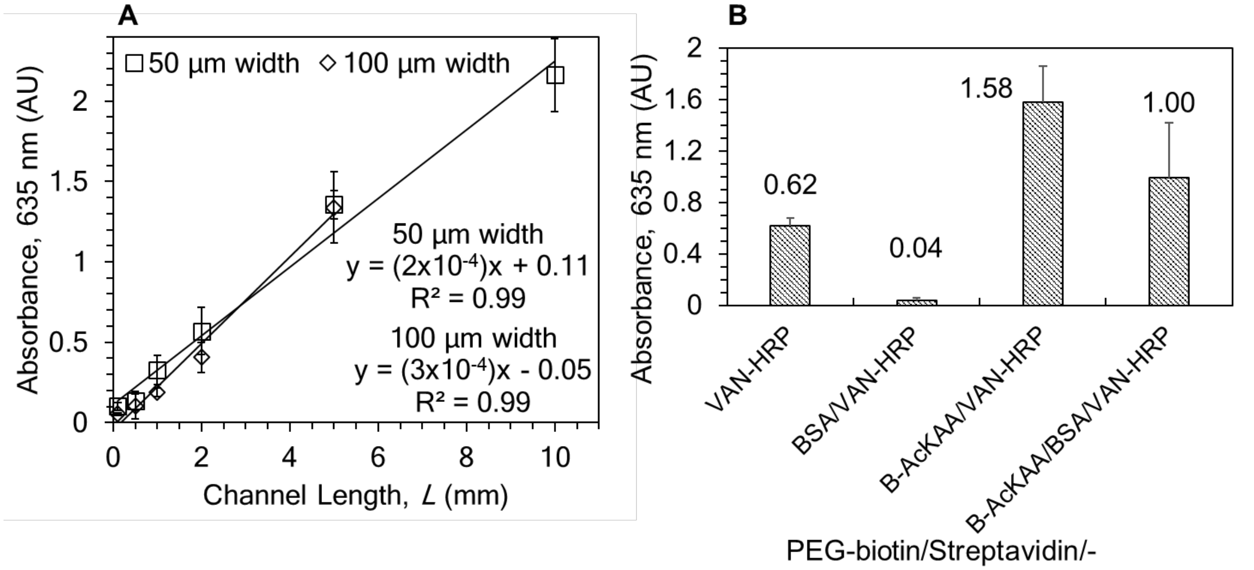

Figure *S*1 Absorbance of the TMB model-dye (methyl green) in the optofluidic sensing system as a function of optical waveguide length (0.1-10 mm) and width (50-100 μm) (A); and absorbance of the TMB end-product at 635 nm due to Vancomycin-HRP (VAN-HRP) activity at 0.7 μM in the microneedle base surfaces where bovine serum albumin (BSA) was used for surface blocking to prevent non-specific binding of VAN-HRP (B). For all measurements, n = 4 and error bars represent standard deviations.
